# Individual and Co Transport Study of Titanium Dioxide NPs and Zinc Oxide NPs in Porous Media

**DOI:** 10.1371/journal.pone.0134796

**Published:** 2015-08-07

**Authors:** Jyoti Kumari, Ankita Mathur, A. Rajeshwari, Arthi Venkatesan, Satyavati S, Mrudula Pulimi, Natarajan Chandrasekaran, R. Nagarajan, Amitava Mukherjee

**Affiliations:** 1 Centre for Nanobiotechnology, VIT University, Vellore, Tamil Nadu, India; 2 Department of Microbiology and Cell Biology, IISc, Bangalore, Karnataka, India; 3 Department of Chemical Engineering, IIT Madras, Chennai, India; Institute for Materials Science, GERMANY

## Abstract

The impact of pH and ionic strength on the mobility (individual and co-transport) and deposition kinetics of TiO_2_ and ZnO NPs in porous media was systematically investigated in this study. Packed column experiments were performed over a series of environmentally relevant ionic strengths with both NaCl (0.1−10 mM) and CaCl_2_ (0.01–0.1mM) solutions and at pH 5, 7, and 9. The transport of TiO_2_ NPs at pH 5 was not significantly affected by ZnO NPs in solution. At pH 7, a decrease in TiO_2_ NP transport was noted with co-existence of ZnO NPs, while at pH 9 an increase in the transport was observed. At pH 5 and 7, the transport of ZnO NPs was decreased when TiO_2_ NPs was present in the solution, and at pH 9, an increase was noted. The breakthrough curves (BTC) were noted to be sensitive to the solution chemistries; the decrease in the breakthrough plateau with increasing ionic strength was observed under all examined pH (5, 7, and 9). The retention profiles were the inverse of the plateaus of BTCs, as expected from mass balance considerations. Overall, the results from this study suggest that solution chemistries (ionic strength and pH) are likely the key factors that govern the individual and co-transport behavior of TiO_2_ and ZnO NPs in sand.

## Introduction

Various studies have shown that TiO_2_ NPs is toxic to organisms like rats, algae, microbes, invertebrates, and fish. The yearly production of TiO_2_ NPs is expected to expand to ∼2.5 million metric tons by 2025 in the United States alone [1]. Due to their extensive production and use in household and industrial commodities, some of the TiO_2_ NPs utilized have been discharged into the natural aquatic environment directly or indirectly. Additionally, reports suggest that 10–100 mg/L of Ti is present in municipal wastewater treatment plant effluents [2]. Thus, mechanisms controlling the transport and deposition of these nanomaterials in porous media are critical to understand in order to thoroughly evaluate the mobility and persistence of these materials in aquatic environments as their bioavailability and toxicity will inevitably be governed by their fate and transport in the environment [3, 4].

The most propitious nanoparticles being identified are ZnO NPs because of their extensive utilization in various industries (e.g. optical and chemical) [5, 6, 7, 8, 9, 10] and commercialized products (e.g. cosmetics) [7, 11, 12]. Yearly production of ZnO NPs has grown over 30, 000 tons/year globally [13] mostly due to their diverse applications [14, 15, 16]. Several recent reports have explained that ZnO NPs can cause apoptosis to mammalian cells [16, 17, 18, 19], acute toxic effect and [20, 21], phytotoxicity to plant roots [22, 23], etc. These applications potentially lead to direct and indirect release of ZnO NPs in the aquatic environment. The potential environmental and health risks of nanoparticles are greatly dependent on their fate and behavior in the natural environment in terms of exposure scenarios [24]. Upon discharge, nanoparticles will get released into soil and groundwater [3]. In order to quantitatively assess the potential ecological risk, it is extremely imperative to understand the transport and distribution of ZnO NPs in natural porous media system.

Substantial studies have been done previously on transport and retention of TiO_2_ NPs and ZnO NPs in porous media [25]. These studies mainly concentrate on the transport and retention of one kind of nanomaterial, either TiO_2_ or ZnO NPs in porous media. Owing to their various applications in a broad variety of fields, there is increased probability for the conjoint entry of different classes of nanomaterials in nature. Various studies on the co transport of materials like clay, virus, and bacteria, (that did not particularly deal with engineered nano materials) have been reported in recent years, and it was shown that co transport behavior was more complicated than that of individual constituents, stipulating the necessity to evaluate the co transport of nanoparticles [26, 27].

Therefore, the aim of this work was to study the individual and co transport behavior of TiO_2_ and ZnO NPs, which has a wide range of applications under a series of ionic strengths (0.1–10 mM NaCl, 0.01–0.1 mM CaCl_2_) and pH (5,7, and 9). The breakthrough curves (BTCs) and retention profiles of both TiO_2_ NPs and ZnO NPs were compared with individual transport study. The current study showed the effect of co transport of nanoparticles at different pH and ionic strengths. The packed column experiments were performed over a series of environmentally relevant ionic strength in both NaCl (0.1−10 mM) and CaCl_2_ (0.01–0.1 mM) and at pH (5, 7, and 9). Transport of only TiO_2_ NPs was observed to be relatively low at pH 5, whereas, higher BTCs were observed at pH 7 and 9. However, the transport of TiO_2_ NPs in the presence of ZnO NPs was almost similar to those without ZnO at pH 5, but at pH 7, the presence of ZnO NPs showed a decrease in TiO_2_ NP transport as indicated by the BTCs. The co presence of ZnO NPs in suspensions increased the transport of TiO_2_ NPs in sand at pH 9. On the other hand, ZnO NP transport in sand at pH 5 and 7 was less because of the attractive force between sand and NPs. At pH 9, the transport of ZnO NPs increased in sand because of similar zeta potential values for sand and NPs. ZnO NPs in the co presence of TiO_2_ NPs in suspension showed a decrease in transport than that of those without TiO_2_ NPs at pH 5. However, at pH 7, the co presence of TiO_2_ NPs in ZnO NP suspension unveils increase in transport of NPs. The transport in co presence of TiO_2_ NPs at pH 9 showed an increase in ZnO NP transport under all examined ionic strength. The retention profiles were the inverse of the plateaus of BTCs, as expected from mass balance considerations. The results from this study strongly suggest that the solution chemistries (ionic strength and pH) are likely to play an important role in the individual and co transport characteristics of TiO_2_ and ZnO NPs in sand. This study is the first of its kind to evaluate the co transport of two very important commercially used nanomaterials, TiO_2_ and ZnO NPs, which possess substantial environmental risks also.

## Materials and Methods

### Preparation of TiO_2_ NPs and ZnO NP suspension

Titanium dioxide nanoparticles powder (TiO_2_ anatase, less than 25 nm in diameter, purity greater than 99.9%) and ZnO NPs (less than 100 nm) were purchased from Sigma-Aldrich Corp. TiO_2_ nanoparticle stock suspension (100 mg/ L) was prepared by suspending TiO_2_ nanopowder in Milli-Q water and sonicating using an ultrasonic processor (Sonics., USA) of 130 W, for 10 min. The aqueous suspensions of ZnO NPs were prepared by suspending ZnO nanopowder in Milli-Q water and sonicating in a bath sonicator for 10 min at 530 W, for 20 min (Crest Ultrasonics). As the nanoparticles, sand, and other chemicals used in the experiment were procured, no specific permissions were required from anywhere. All the tests were carried out within the laboratory, and due care was taken not to contaminate the environment.

For both individual nanoparticles transport and nanoparticles co transport experiments, the influent concentrations of TiO_2_ NPs and ZnO NP suspensions were maintained to be 10 and 5 mg/L, respectively. The ionic strengths of nanoparticles suspensions ranged from 0.1 to 10 mM in NaCl solutions and 0.01 to 0.1 mM in CaCl_2_ solutions. The suspension pH was set to be 5, 7, and 9 by adjusting with 0.1 M HCl or 0.1 M NaOH. The zeta potentials and particle sizes of nanoparticles under these conditions were measured using Brookhaven Instruments Corporations, USA. The measurements were performed at room temperature (25°C) and repeated several times.

### Porous Media

Quartz sand (CAS Number 14808-60-7, Sigma-Aldrich) was used as the porous media for all nanoparticle transport experiments. The protocol for cleaning the sand was followed as explained by Li et al. [28]. The zeta potentials of the crushed quartz sand were also measured in NaCl/ CaCl_2_ solutions under the experimental conditions.

### Column Experiments

The cylindrical glass columns (10-cm long and 2-cm inner diameter) were wet-packed with cleaned quartz sand. Briefly, prior to packing, the cleaned quartz sand was rehydrated by boiling in Milli-Q water for at least 1 h. After the rehydrated quartz sand was cooled, the columns were packed by adding wet quartz sand in small increments (∼ 1 cm) with mild vibration of the column so as to reduce any layering or air entrapment [29]. The gravimetrically measured bed porosity was approximately 0.40.The approximate porosities of TiO_2_ NPs, ZnO NPs, and sand were 0.0065, 0.038, and 0.00019, respectively ((Micromeritics, TriStar ш, USA). The temperature was 27±5° C throughout the experiment. The absorbance of ZnO and TiO_2_ nanoparticles were taken at 370 nm and 330 nm, respectively. The dielectric constant of sand column was estimated according to the formula: εr = [∑Vi(εri)0.5]^2^ [30]. The dielectric constant values for TiO_2_ and ZnO NPs without sand were 100 and 3.68, respectively[31,32]. On the other hand, the dielectric constant values for TiO_2_ and ZnO NPs with sand were 400.96 and 399.99.

The columns were pre-equilibrated with at least ten pore volumes of NaCl /CaCl_2_ solutions maintained at a desired ionic strength and pH after packing. Following pre-equilibration, three pore volumes of nanoparticle suspensions were passed into the column, followed by recovery with five pore volumes of NaCl/CaCl_2_ solution at the same ionic strength and pH. The solutions were injected into the columns in a down-flow mode using a peristaltic pump. During the column experiments, the input nanoparticle suspensions were periodically sonicated to evade any settlement of nanoparticles and to sustain the stability of the suspension at desired solution chemistries. The transport experiments were conducted at three ionic strengths (0.1, 1, and 10 mM) in NaCl and (0.01, 0.05, and 0.1 mM) CaCl_2_ solutions, at three pH conditions (pH 5, 7, and 9). The pore water velocity of all experiments was set to 8 m/day (0.69 mL/min) in order to represent the fluid velocities in coarse aquifer sediments [33,34].

The column effluent samples were collected (∼15 mL) in centrifuge tubes at pre calculated time intervals. Following the transport experiment, the sand was taken out from the column under gravity and disjoint into 10 segments (each 1 cm). To release the nanoparticles from quartz sand, approximately 5−10 mL of 0.1 M NaOH/CaCl_2_ solution was added to each sediment segment, and the mixture was continuously shaken at 300 rpm overnight, and then manually shaken vigorously for a few seconds [35]. The effluent samples and supernatant samples from the recovery of retained TiO_2_ NPs were analyzed using a UV spectrophotometer (U2910, Hitachi, Japan), whereas the corresponding samples for ZnO NPs were evaluated by UV spectrophotometer (U2910, Hitachi, Japan). The area under the breakthrough elution curve was integrated to provide the percentage of nanoparticles that evacuated the column. The percentage of nanoparticles retrieved from the sediment was obtained by adding the quantity of nanoparticles retrieved from all segments of the sediment and dividing by the total amount of nanoparticles injected. The sum of the percentages of retained particles and particles that exited the column represented the overall recovery (mass balance) of nanoparticles. The mass recovery of nanoparticles for each experiment is provided in the S4–S7 Tables.

### Characterization of transport and transported material

#### Transmission electron microscopy (TEM)

Size and morphology of individual nanoparticles (TiO_2_ and ZnO NPs) before and after transport in porous media were characterized using transmission electron microscopy (Technai G2 spirit biotwin (120kv), FEI Company). Samples were collected in 15-ml centrifuge tube before and after nanoparticle (TiO_2_ and ZnO NP) transport in porous media at pH 5, 7 and 9 at an ionic strength of 10 mM NaCl. The aerated samples (60°C, 30 min) were then deposited onto the lacey carbon-coated copper grid and subjected to TEM analysis.

#### X-ray diffraction patterns

To characterize the TiO_2_ NPs, ZnO NPs, and sand, X-ray diffraction analysis was carried out using the X-ray Diffraction spectrophotometer (Bruker, D8 Advance, Germany). TiO_2_ NPs, ZnO NPs, and sand powder were grinded to a make a fine powder, and a uniform smear was prepared on a glass slide. The prepared sample was packed into a sample container for analysis. The crystalline structure of TiO_2_ NPs, ZnO NPs, and sand were determined with the diffraction patterns obtained.

#### Fourier transform infrared spectroscopy

The functional interaction between the transport and transported material was investigated using FTIR analysis. The nanoparticle samples before and after transport through the porous media were collected and used for analysis.

### Statistical analysis

All experiments were carried out in triplicates and the data are given as mean ± standard error. The data were processed using Student’s t-test with a p value < 0.05.

### Conductivity Measurement

Conductivity was evaluated for both the nanoparticles (ZnO NPs and TiO_2_ NPs) under a series of ionic strengths in both NaCl (0.1, 1, and 10 mM) and CaCl_2_ (0.01, 0.05, and 0.1 mM) solutions at three different pH conditions, 5, 7, and 9, before and after transport through the porous media. After transport, the samples were collected, and the conductivity was measured using conductivity meter (ELICO, CM 180).

## Results and Discussion

### Effect of ZnO NPs on TiO_2_ NP Transport in sand column

**Fig 1:**
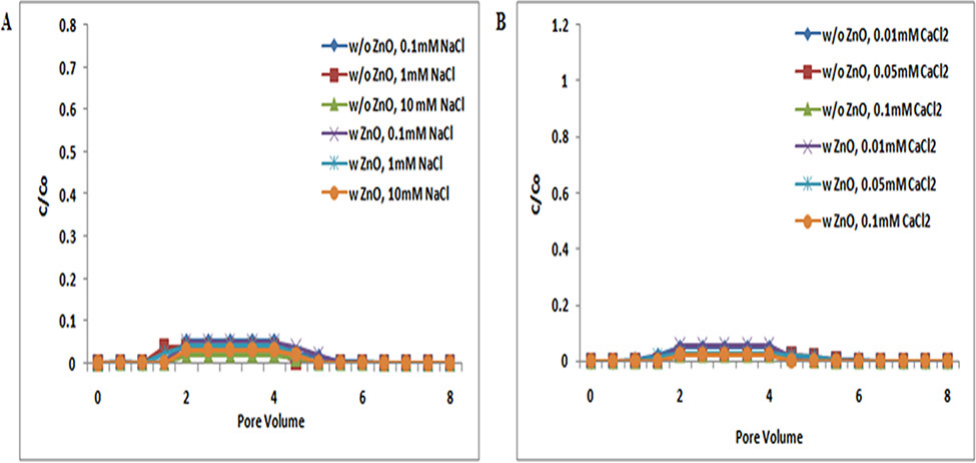
Breakthrough curves of TiO_2_ NPs at pH 5 (with and without ZnO NPs). Breakthrough curves of TiO_2_ NPs in presence and absence of ZnO NPs in sand. In suspensions at 0.1, 1, and 10 mM ionic strengths in NaCl solutions and 0.01, 0.05, and 0.1 mM CaCl_2_ solutions at pH 5. Replicate experiments were performed under all conditions (n ≥ 2). No effect was seen on TiO_2_ NP transport in co presence of ZnO NPs in porous media.

**Fig 2:**
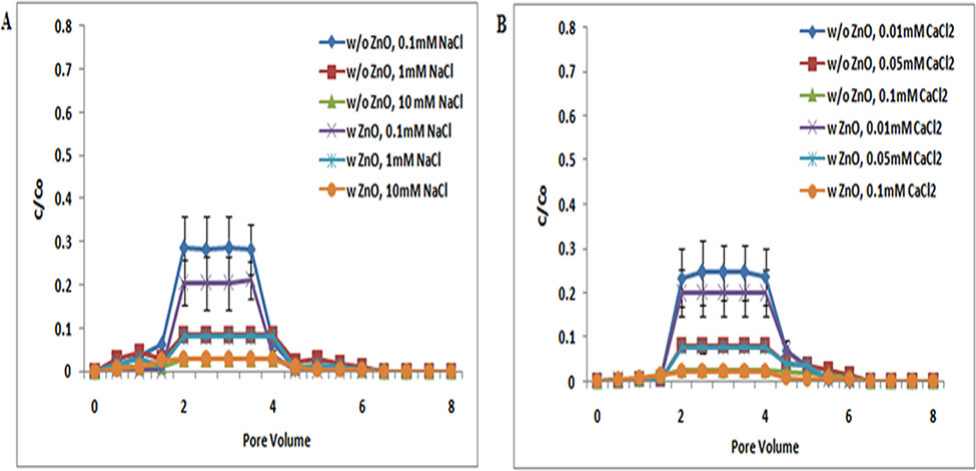
Breakthrough curves of TiO_2_ NPs at pH 7 (with and without ZnO NPs). Breakthrough curves of TiO_2_ NPs in presence and absence of ZnO NPs in sand. In suspensions at 0.1, 1, and 10 mM ionic strengths in NaCl solutions and 0.01, 0.05, and 0.1 mM CaCl_2_ solutions at pH 7. Replicate experiments were performed under all conditions (n ≥ 2).In presence of ZnO NPs in TiO_2_ NP suspension showed decrease in transport of TiO_2_ NPs in porous media.

**Fig 3:**
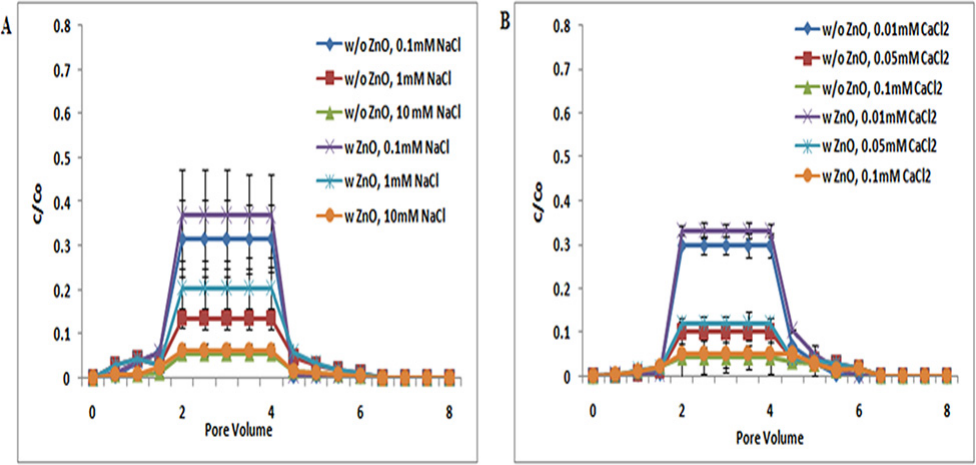
Breakthrough curves of TiO_2_ NPs at pH 9 (with and without ZnO NPs). Breakthrough curves of TiO_2_ NPs in presence and absence of ZnO NPs in sand. In suspensions at 0.1, 1, and 10 mM ionic strengths in NaCl solutions and 0.01, 0.05, and 0.1 mM CaCl_2_ solutions at pH 9. Replicate experiments were performed under all conditions (n ≥ 2). At this pH there was increase in transport of TiO_2_ NPs in porous media in presence of ZnO NPs in suspension.

The transport of TiO_2_ NPs in absence and presence of ZnO NPs in sand was examined under a series of ionic strengths in both NaCl (0.1, 1, and 10 mM) and CaCl_2_ (0.01, 0.05, and 0.1 mM) solutions at three different pH conditions, 5, 7, and 9. The breakthrough curves of TiO_2_ NPs without ZnO NPs are presented in Figs 1, 2 and 3. The zeta potential of TiO_2_ NPs was positive; however, the zeta potential of bare sand was negative in both the NaCl (0.1, 1, and 10 mM) and CaCl_2_ (0.01, 0.05, and 0.1 mM) solutions at pH 5 (S1 and S3 Tables). Therefore, an attractive electrostatic interaction was expected between TiO_2_ NPs and sand under the examined pH 5 for all ionic strengths. As a result, under all ionic strengths [NaCl (0.1, 1, and 10 mM); CaCl_2_ (0.01, 0.05, and 0.1 mM)] at pH 5, TiO_2_ NPs were retained in sand and very less amount of NPs had eluted out. This indicates that the delivery of TiO_2_ NPs without ZnO NPs was intensely dependent on pH. Previous reports by Chowdhary et al. and Solvitch et al. supported our findings as these studies also suggested that TiO_2_ NPs have different zeta potential values at different pH, and thus at disparate pH, the transport of TiO_2_ NPs varies [36, 37]. Though size and zeta potential for the TiO_2_ NPs were heterogeneous at different solution conditions, the BTC curves of TiO_2_ NPs were similar under all inspected ionic strengths (S1 Table). These results demonstrate that the impact of ionic strength on TiO_2_ transport was least at pH 5. A previous report on the cotransport of TiO_2_ NPs and C60 NPs by Cai, L et al. also validated that the impact of ionic strength on TiO_2_ NP transport was least at pH 5 [38]. Additionally, the BTCs of TiO_2_ NPs in presence of ZnO NPs at pH 5 were nearly equivalent to those without ZnO NPs, under all ionic strengths (NaCl/ CaCl_2_) (Fig 1). Therefore, these results clearly demonstrated that there were no significant (p> 0.05) changes in the transport of TiO_2_ NPs in presence of ZnO NPs at pH 5.

Both TiO_2_ NPs and sand have negative zeta potential at pH 7 (S1 and S3 Tables), and so repulsive electrostatic interaction was expected between TiO_2_ NPs and sand under the examined pH 7 and ionic strength [NaCl (0.1, 1, and 10 mM); CaCl_2_ (0.01, 0.05, and 0.1 mM)]. At pH 7, the BTCs obtained for TiO_2_ NPs without ZnO NPs was higher at low ionic strengths. BTCs were sensitive to solution chemistries as indicated by the decrease in breakthrough plateau with increasing ionic strength. The lower BTCs of TiO_2_ NPs with increasing ionic strength, as observed at pH 7, were consistent with the lower negative zeta potentials observed at high ionic strengths, and thus agreed with the classic Derjaguin−Landau−Verwey−Overbeek (DLVO) theory (Figs 1, 2 and 3) [39]. This theory explains the aggregation of aqueous dispersions quantitatively and describes the force between charged surfaces interacting through an aqueous medium. It integrates the effects of Van der Waal’s attraction and electrostatic repulsion due to the so-called double layer of counter ions. Cai L et al. and Chen G et al. also reported that the transport of TiO_2_ increases with increasing ionic strengths, and thus, agreed with DLVO theory [38, 40]. Fang et al. also reported that the variation in pH also altered both the magnitude and separation distance of the repulsive energy barrier in DLVO interaction [39]. Moreover, breakthrough curves in copresence of ZnO NPs at pH 7 showed a decrease in TiO_2_ NP transport. These may be due to charge heterogeneity present in TiO_2_ NPs, ZnO NPs, and sand. However, the results clearly show that there are no significant (p> 0.05) changes in the transport of TiO_2_ NPs in presence of ZnO at pH 7.

At pH 9, the zeta potential values of TiO_2_ NPs and sand are more negative than pH 7. Thus, more repulsive electrostatic interaction was expected between TiO_2_ NPs and sand under the examined pH 9 and all ionic strength [NaCl (0.1, 1, and 10 mM); CaCl_2_ (0.01, 0.05, and 0.1 mM)](S1 and S3 Tables). Breakthrough curve of TiO_2_ NPs at pH 9 was higher than at pH 7. But the BTC plateau in co presence of ZnO NPs was increased at pH 9 (Fig 3). These results showed that, unlike the negligible change of TiO_2_ transport induced by the presence of ZnO NPs at pH 5, the copresence of ZnO NPs in suspensions increased the transport of TiO_2_ NPs in sand at pH 9. ZnO NPs in suspension may compete with TiO_2_ NPs for deposition sites on sand, contributing to the enhanced transport of TiO_2_ NPs in sand at pH 9. However no significant difference (p> 0.05) was noted in transport of individual of TiO_2_ NPs when compared to co-transport of TiO_2_ NPs with ZnO NPs.

The above results demonstrated that pH plays the most important role in the transport and retention of TiO_2_ NPs and ZnO NPs. BTCs of TiO_2_ NPs (Figs 1, 2 and 3) at pH 5 were lower in sand due to the electrostatic attraction between sand and TiO_2_ NPs (Figs 1, 2 and 3). The electrostatic attraction between sand and TiO_2_ NPs at pH 5 has also been reported previously by Cai Li et al. [38]. However, at pH 7 and 9, negatively charged TiO_2_ NPs showed repulsion to negatively charged sand, and thereby a decrease in the retention of TiO_2_ NPs in sand was noted. The negative zeta potential values of sand and TiO_2_ NPs at pH 7 have also been also reported by Dunphy et al. [41]. This observation was true under all ionic strength conditions [NaCl (0.1, 1, and 10 mM); CaCl_2_ (0.01, 0.05, and 0.1 mM)].

### Effect of TiO_2_ NPs on ZnO NP Transport in sand column

The influence of TiO_2_ NP copresence in suspensions on the transport behavior of ZnO NPs was examined. The transport experiments of ZnO NPs in packed sand were performed both in the absence and presence of TiO_2_ NPs at different ionic strength [NaCl (0.1, 1, and 10 mM); CaCl_2_ (0.01, 0.05, and 0.1 mM)] and pH conditions (5, 7, and 9). As shown in S2 and S3 Tables, at pH conditions 5 and 7, the zeta potential values of ZnO NPs were positive, but at pH 9, the zeta potential of ZnO NPs was negative. Sand was negatively charged under all examined ionic strength conditions and pH (S3 Table).

**Fig 4:**
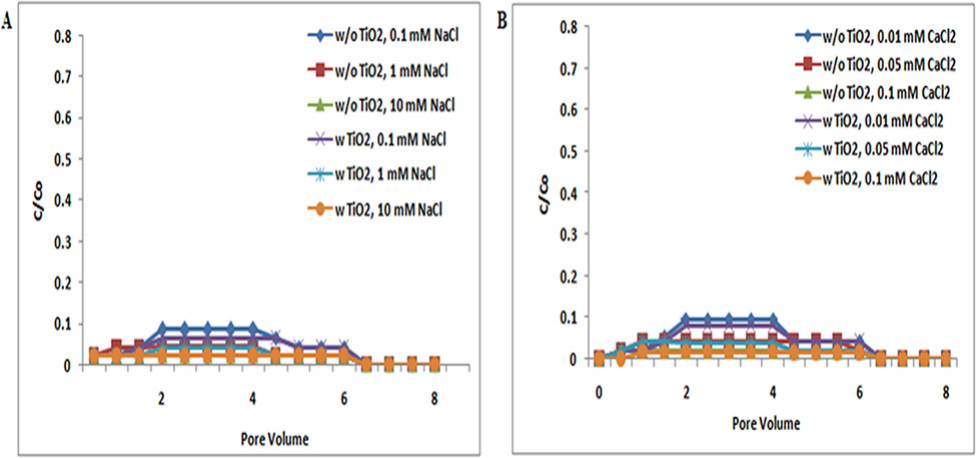
Breakthrough curves of ZnO NPs at pH 5 (with and without TiO_2_ NPs). Breakthrough curves of ZnO NPs in presence and absence of TiO_2_ NPs in sand. In suspensions at 0.1, 1, and 10 mM ionic strengths in NaCl solutions and 0.01, 0.05, and 0.1 mM CaCl_2_ solutions at pH 5. Replicate experiments were performed under all conditions (n ≥ 2). In presence of TiO_2_ NPs in suspension of ZnO NPs showed decrease in transport of TiO_2_ NPs in porous media.

**Fig 5:**
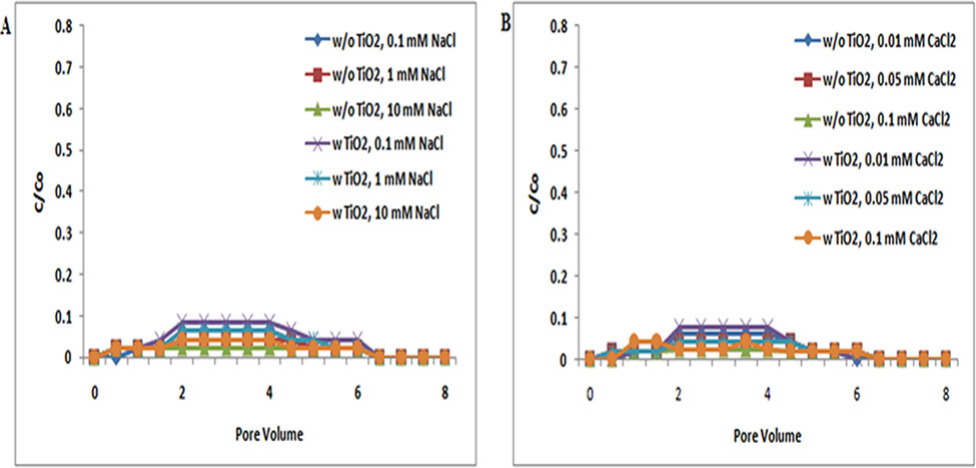
Breakthrough curves of ZnO NPs at pH 7 (with and without TiO_2_ NPs). BTCs of ZnO NPs in presence and absence of TiO_2_ NPs sand. In suspensions at 0.1, 1, and 10 mM ionic strengths in NaCl solutions and 0.01, 0.05, and 0.1 mM CaCl_2_ solutions at pH 7. Replicate experiments were performed under all conditions (n ≥ 2). In presence of TiO_2_ NPs in suspension of ZnO NPs showed increase in transport of TiO_2_ NPs in porous media.

**Fig 6:**
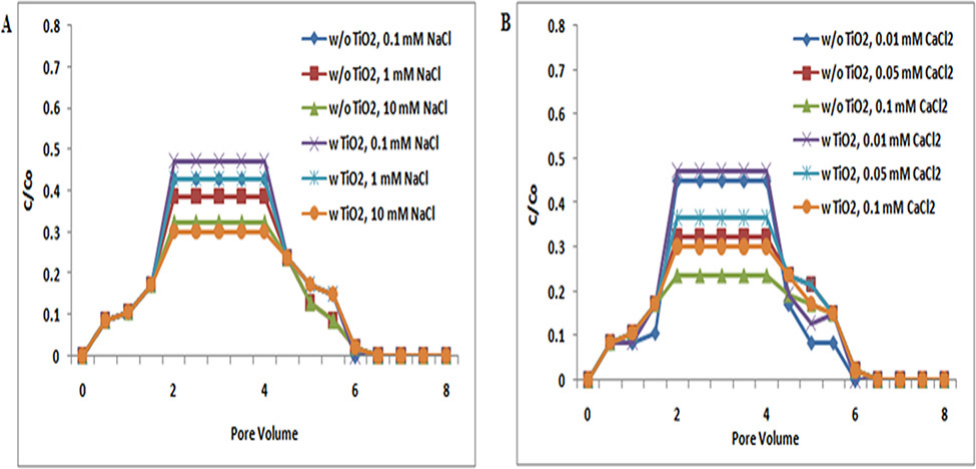
Breakthrough curves of ZnO NPs at pH 9 (with and without TiO_2_ NPs). BTCs of ZnO NPs in presence and absence of TiO_2_ NPs sand. In suspensions at 0.1, 1, and 10 mM ionic strengths in NaCl solutions and 0.01, 0.05, and 0.1 mM CaCl_2_ solutions at pH 9. Replicate experiments were performed under all conditions (n ≥ 2). In presence of TiO_2_ NPs in suspension of ZnO NPs showed increase in transport of TiO_2_ NPs in porous media.

The electrostatic interaction between ZnO NPs and quartz sand was attractive at both pH 5 and 7 [41]. At pH 5 and 7, most of the ZnO NPs are retained in the column, and very less amount of ZnO NPs had eluted out. The apparent breakthrough of ZnO NPs in sand was observed under all examined ionic strength conditions [NaCl (0.1, 1, and 10 mM); CaCl_2_ (0.01, 0.05, and 0.1 mM)] at both pH 5 and 7 (Figs 4, 5 and 6).

Additionally, ZnO NPs in copresence of TiO_2_ NPs in suspension showed decrease in BTC plateau when compared with samples without TiO_2_ NPs under all examined ionic strength conditions at pH 5 (Fig 4). From the above results, it was demonstrated that, the copresence of TiO_2_ NPs in ZnO NP suspension at pH 5 decreases the transport of ZnO NPs in sand under all examined ionic strength conditions [NaCl (0.1, 1, and 10 mM); CaCl_2_ (0.01, 0.05, and 0.1 mM)].

However, the copresence of TiO_2_ NPs with ZnO NP suspension at pH resulted in the increase of BTC curve. The zeta potential values of both ZnO NPs and sand were negative at pH 7 under all examined ionic strength conditions [NaCl (0.1, 1, and 10 mM); CaCl_2_ (0.01, 0.05, and 0.1 mM)] (S2 and S3 Tables). Repulsive electrostatic force was expected between ZnO NPs and sand under-examined pH 7 and all ionic strength [NaCl (0.1, 1, and 10 mM); CaCl_2_ (0.01, 0.05, and 0.1 mM)]. BTCs were sensitive to solution chemistries, as indicated by the increase of the breakthrough plateau with increasing ionic strength. However, the difference in individual transport and cotransport was not statistically significant (p> 0.05) at pH 5 and 7.

At pH 9, the BTCs of ZnO NPs obtained was higher at low ionic strength. The lower BTCs of ZnO NPs with increasing ionic strength, as observed at pH 9, were consistent with the less negative zeta potentials observed at high ionic strength, and thus, the results agreed with the classic Derjaguin−Landau−Verwey−Overbeek (DLVO) theory. However, the breakthrough curve in copresence of TiO_2_ at pH 9 showed an increase in ZnO NP transport under all examined ionic strength [NaCl (0.1, 1, and 10 mM); CaCl_2_ (0.01, 0.05, and 0.1 mM)](Fig 6). But, the results demonstrated that there are no significant (p> 0.05) changes in the transport of ZnO NPs in presence of TiO_2_ NPs at pH 7.

The results from the above studies showed that the co presence of both the NPs (TiO_2_ and ZnO) affected the transport of each other at different pH. At pH 5 and 7, the positive charge of ZnO NPs causes the increase in attractive force between sand and NPs, and therefore, the BTCs were decreased. Moreover, the transport of ZnO NPs increased in sand at pH 9 because of the similar zeta potential. The positive zeta potential of ZnO NPs at pH 5 and 7 and negative zeta potential at pH 9 have been reported by Kanel et al. [42].These observations were true under all ionic strength conditions [NaCl (0.1, 1, and 10 mM); CaCl_2_ (0.01, 0.05, and 0.1 mM)]. The breakthrough rates for all the transport experiment were observed at a pore volume of 2±0.5.

### Column retention profile

#### Effect of ZnO NPs on the retention of TiO_2_ NPs

The retention profiles of TiO_2_ NPs and ZnO NPs under all ionic strength conditions, both for the individual and cotransport experiments, were studied to check if cotransport of TiO_2_ NPs and ZnO NPs would affect the distribution of nanoparticles retained in quartz sand. As anticipated from mass balance data, the retention profile obtained was the reverse of the BTC curves.

**Fig 7:**
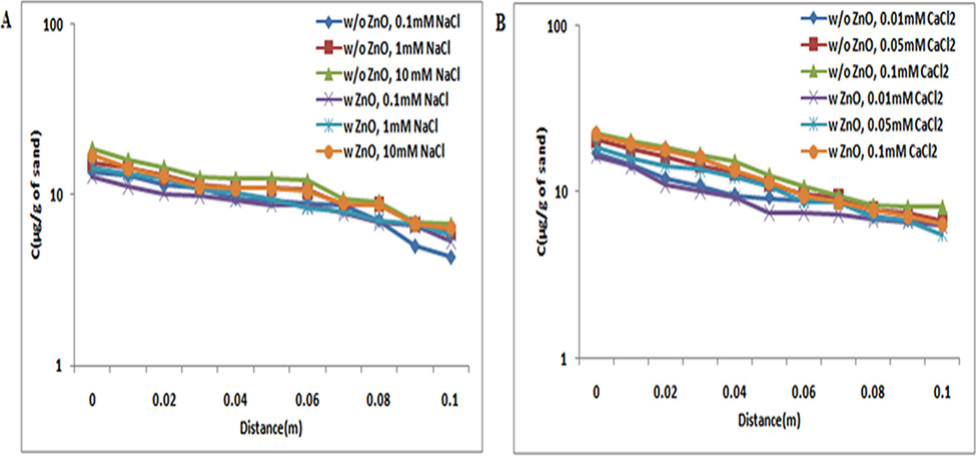
Retention profile of TiO_2_ NPs at pH 5 (with and without ZnO NPs). Retention graph of TiO_2_ NPs in presence and absence of ZnO NPs sand. In suspensions at 0.1, 1, and 10 mM ionic strengths in NaCl solutions and 0.01, 0.05, and 0.1 mM CaCl_2_ solutions at pH 5. Replicate experiments were performed under all conditions (n ≥ 2). Similar retention profile was observed in both individual (TiO_2_ NPs) and co transport experiment (TiO_2_ NPs and ZnO NPs).

**Fig 8:**
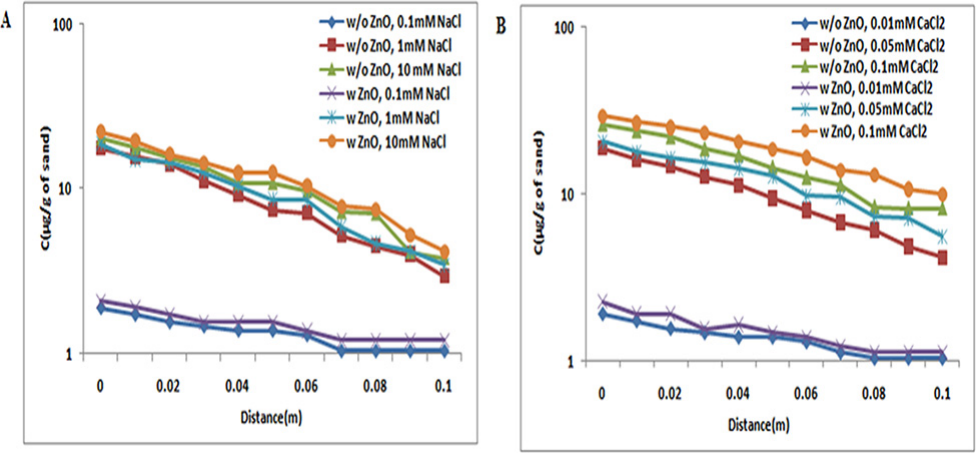
Retention profile of TiO_2_ NPs at pH 7 (with and without ZnO NPs). Retention graph of TiO_2_ NPs in presence and absence of ZnO NPs sand. In suspensions at 0.1, 1, and 10 mM ionic strengths in NaCl solutions and 0.01, 0.05, and 0.1 mM CaCl_2_ solutions at pH 7. Replicate experiments were performed under all conditions (n ≥ 2). There was increase in retention profile of TiO_2_ NPs in co transport experiment (TiO_2_ NPs and ZnO NPs). In presence of ZnO NPs in suspension of TiO_2_ NPs showed increase in retention of TiO_2_ NPs in porous media.

**Fig 9:**
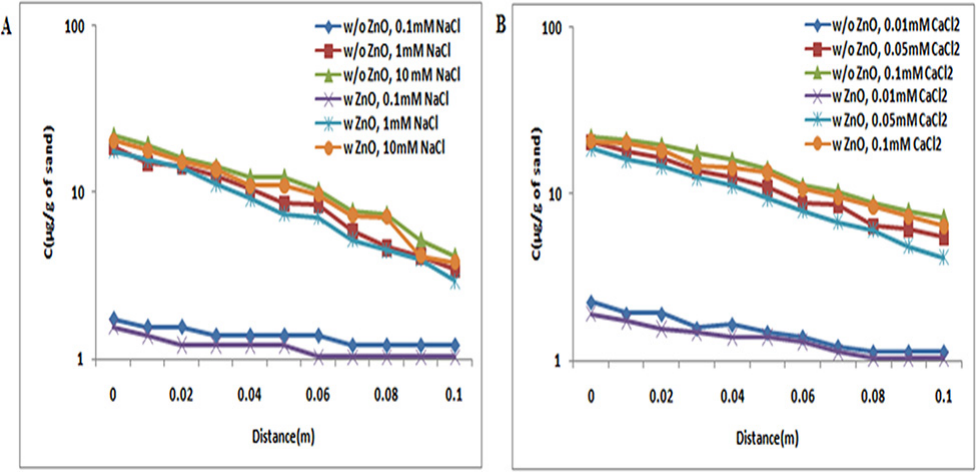
Retention profile of TiO_2_ NPs at pH 9 (with and without ZnO NPs). Retention graph of TiO_2_ NPs in presence and absence of ZnO NPs sand. In suspensions at 0.1, 1, and 10 mM ionic strengths in NaCl solutions and 0.01, 0.05, and 0.1 mM CaCl_2_ solutions at pH 9. Replicate experiments were performed under all conditions (n ≥ 2). At pH 9 also in presence of ZnO NPs in suspension of TiO_2_ NPs showed increase in retention of TiO_2_ NPs in porous media.

At pH 5, under all examined ionic strength conditions in the absence of ZnO NPs in suspensions, the retained concentration of TiO_2_ NPs in quartz sand decreased log−linearly with increase in distance. Retained concentrations of TiO_2_ NPs in quartz sand decreased non-exponentially with distance. However, the retention profile of TiO_2_ NPs in presence of ZnO NPs was similar to the profile acquired in the absence of ZnO NPs (Figs 7, 8 and 9). This study demonstrated that the copresence of ZnO NPs in suspensions did not affect the retention of TiO_2_ NPs in sand at pH 5.

At pH 7, the BTC plateau was decreasing with the increase in ionic strengths (NaCl and CaCl_2_). Figs 7, 8 and 9 shows that TiO_2_ NP retention was delegate to the solution ionic strength. Under all ionic strength conditions [NaCl (0.1, 1, and 10 mM); CaCl_2_ (0.01, 0.05, and 0.1 mM)] at pH 7, the retention being detected near the column inlet was highest. Additionally, in the presence of ZnO NPs, the retained concentration of TiO_2_ NPs was higher relative to the concentration retained in the absence of ZnO NPs.

At pH 9 under all ionic strength conditions [NaCl (0.1, 1, and 10 mM); CaCl_2_ (0.01, 0.05, and 0.1 mM)], the retention being detected near the column inlet was highest (Figs 7, 8 and 9). The retained concentration of TiO_2_ NPs were higher in comparison to retention observed in the absence of ZnO NPs. These results indicated that at pH 9 (unfriendly conditions), the copresence of ZnO NPs in suspensions increases the retention of TiO_2_ NPs in sand. A close inspection of retention profiles of TiO_2_ NPs in the presence of ZnO NPs in suspensions versus those in the absence of ZnO NPs displayed that under all ionic strength conditions [NaCl (0.1, 1, and 10 mM); CaCl_2_ (0.01, 0.05, and 0.1 mM)], the retention profiles of TiO_2_ in the presence of ZnO NPs were lower to those without ZnO NPs in suspension.

The retention profiles were the inverse of the plateaus of BTCs, as expected from mass balance considerations (S4 and S5 Tables). Retention of TiO_2_ NPs in sand at pH 5 was more because of the presence of opposite charges on sand and TiO_2_ NPs. Previous reports also showed that retention of TiO_2_ NPs at pH 5 was higher because at this particular pH, the positively charged TiO_2_ NPs tend to attract the negatively charged sand [38]. Moreover, at pH 7 and 9 the retention of TiO_2_ NPs was less in sand due to presence of similar zeta potential in sand and TiO_2_ NPs. The negative charge of TiO_2_ NPs and sand at pH 7 have also been reported by Han et al. [35]. This observation was true under all ionic strength conditions [NaCl (0.1, 1, and 10 mM); CaCl_2_ (0.01, 0.05, and 0.1 mM)].

#### Effect of TiO_2_ NPs in the retention of ZnO NPs

**Fig 10:**
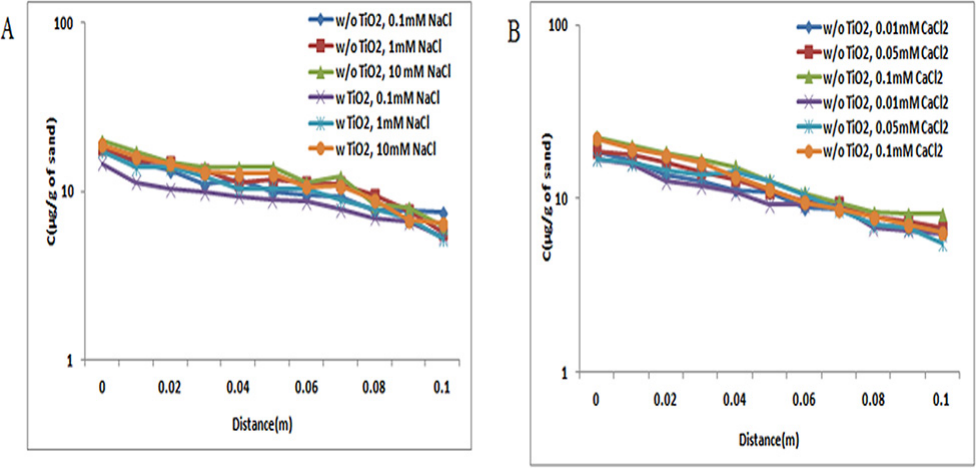
Retention profile of ZnO NPs at pH 5 (with and without TiO_2_ NPs). Retention graph of ZnO NPs in presence and absence of TiO_2_ NPs sand. In suspensions at 0.1, 1, and 10 mM ionic strengths in NaCl solutions and 0.01, 0.05, and 0.1 mM CaCl_2_ solutions at pH 5. Replicate experiments were performed under all conditions (n ≥ 2). In presence of TiO_2_ NPs in suspension of ZnO NPs showed increase in retention of ZnO NPs in porous media.

**Fig 11:**
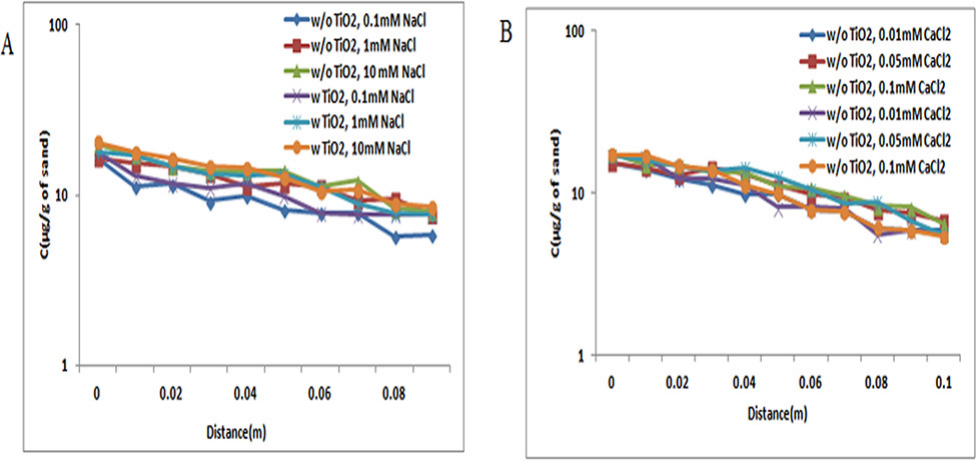
Retention profile of ZnO NPs at pH 7 (with and without TiO_2_ NPs). Retention graph of ZnO NPs in presence and absence of TiO_2_ NPs sand. In suspensions at 0.1, 1, and 10 mM ionic strengths in NaCl solutions and 0.01, 0.05, and 0.1 mM CaCl_2_ solutions at pH 7. Replicate experiments were performed under all conditions (n ≥ 2). In presence of TiO_2_ NPs in suspension of ZnO NPs showed decrease in retention of ZnO NPs in porous media.

**Fig 12:**
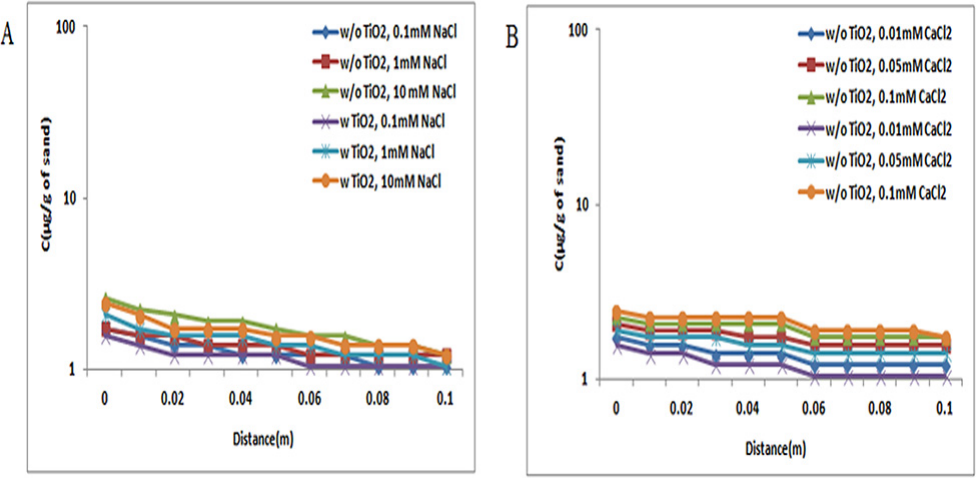
Retention profile of ZnO NPs at pH 9 (with and without TiO_2_ NPs). Retention graph of ZnO NPs in presence and absence of TiO_2_ NPs sand. In suspensions at 0.1, 1, and 10 mM ionic strengths in NaCl solutions and 0.01, 0.05, and 0.1 mM CaCl_2_ solutions at pH 9. Replicate experiments were performed under all conditions (n ≥ 2). In presence of TiO_2_ NPs in suspension of ZnO NPs showed decrease in retention of ZnO NPs in porous media.

The retention profiles of ZnO NPs in presence and absence of TiO_2_ NPs were evaluated under three different pH conditions, 5, 7, and 9, and a series of ionic strengths [NaCl (0.1, 1, and 10 mM) and CaCl_2_ (0.01, 0.05, and 0.1 mM)]. At pH 5, the BTC curves of ZnO NPs were very low due to the opposite charge present between sand and ZnO NPs. So, the retention profile of ZnO NPs in absence of TiO_2_ NPs was very high as shown in Figs 10, 11 and 12. The retention profile of ZnO NPs in the presence of TiO_2_ NPs was higher in sand.

At pH 7, the concentrations of ZnO NPs retained in sand alone were more than that of ZnO NPs copresent with TiO_2_ NPs in the suspensions (Figs 10, 11 and 12). These observations showed that the copresence of TiO_2_ NPs in suspension decreased the deposition of ZnO NPs in sand under all examined conditions. A close comparison of the retention profiles of ZnO NPs in the presence of TiO_2_ NPs versus those in the absence of TiO_2_ NPs showed that under all examined conditions, the decreased retention of ZnO NPs induced by the copresence of TiO_2_ NPs in the suspension occurred across the entire column. Moreover, the shapes of the retention profiles of ZnO NPs in the presence of TiO_2_ NPs were similar to those in the absence of TiO_2_ NPs in suspensions.

In addition, under the examined pH 9, the concentrations of ZnO NPs retained in sand alone were more than that of ZnO NPs copresent with TiO_2_ NPs in suspensions (Figs 10, 11 and 12). The observation showed that the copresence of TiO_2_ NPs in suspension decreased the deposition of ZnO NPs in sand under all examined conditions. A close comparison of the retention profiles of ZnO NPs in the presence of TiO_2_ NPs versus those in the absence of TiO_2_ NPs showed that under all examined conditions, the decreased retention of ZnO NPs induced by the copresence of TiO_2_ NPs in suspension occurred across the entire column.

The retention profiles were the inverse of the plateaus of BTCs, as expected from the mass balance considerations (S6 and S7 Tables). The retention of ZnO NPs was more in sand (Figs 10 and 11) due to opposite charges on ZnO NPs and sand at pH 5 and 7. Moreover, at pH 9, the retention of ZnO NPs was less in sand, because both sand and ZnO NPs acquire negative charges (Fig 12). Previously stated reports also showed that the zeta potential of ZnO NPs vary with pH i.e. at pH 5 and 7, the zeta potential was positive, and at pH 9, it was negative [42].

### Characterization of transport and transported material

#### Transmission electron microscopy (TEM)

**Fig 13:**
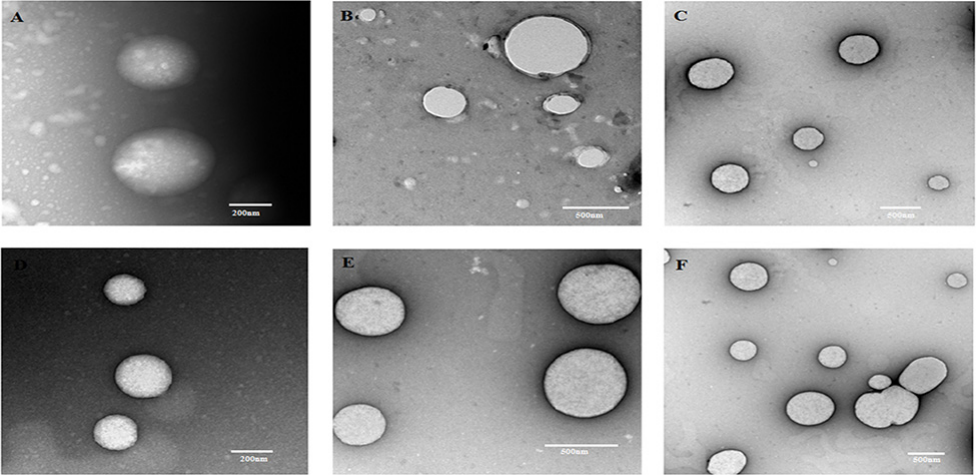
Transmission electron micrograph of TiO_2_ NPs. **A-** Transmission electron microscopic image of TiO_2_ NPs before transport at pH 5. **B-** Transmission electron microscopic image of TiO_2_ NPs after transport at pH 5. **C-** Transmission electron microscopic image of TiO_2_ NPs before transport at pH 7. **D-** Transmission electron microscopic image of TiO_2_ NPs after transport at pH 7. **E-** Transmission electron microscopic image of TiO_2_ NPs before transport at pH 9. **F-** Transmission electron microscopic image of TiO_2_ NPs after transport at pH 9.

**Fig 14:**
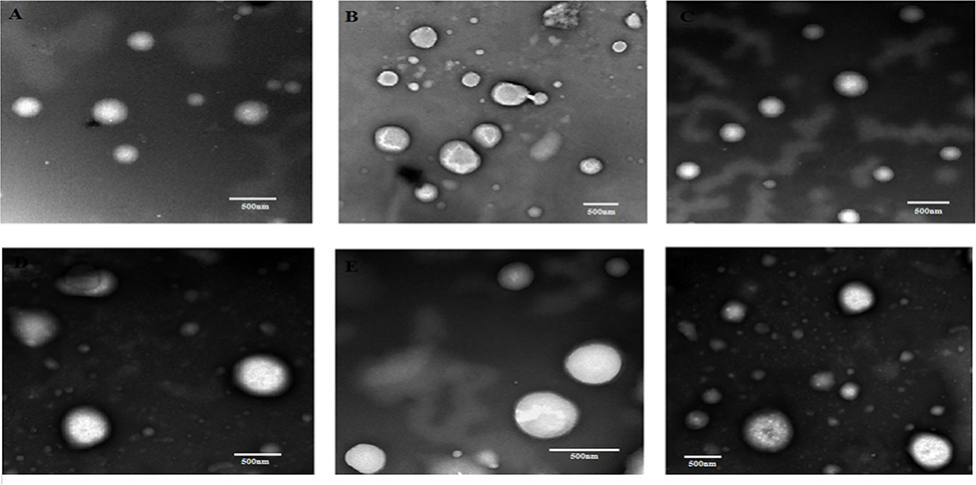
Transmission electron micrograph of ZnO NPs. **A-** Transmission electron microscopic image of ZnO NPs before transport at pH 5. **B-** Transmission electron microscopic image of ZnO NPs after transport at pH 5. **C-** Transmission electron microscopic image of ZnO NPs before transport at pH 7. **D-** Transmission electron microscopic image of ZnO NPs after transport at pH 7. **E-** Transmission electron microscopic image of ZnO NPs before transport at pH 9. **F-** Transmission electron microscopic image of ZnO NPs after transport at pH 9.

The size and shape of both TiO_2_ and ZnO NPs, in presence of 10 mM of NaCl, before and after transport through porous media were examined by transmission electron microscopy (TEM) at three different pH of about 5, 7, and 9. 10 mM of NaCl was chosen based on the high ionic strength. The TEM image of TiO_2_ NPs before transport through porous media shows the uniform distribution of NPs with average sizes of 350, 345, and 363 nm at pH 5, 7, and 9, respectively (Fig 13A, 13C and 13E). The morphology of TiO_2_ NPs was observed to be spherical. Further, the average particle sizes of TiO_2_ NPs at pH 5, 7, and 9 after transport through porous media were noted to be 642, 550, and 489 nm (Fig 13B, 13D and 13F). The sizes of ZnO NPs at pH 5, 7, and 9 were found to be 310, 335, and 333 nm before transport through porous media, and the sizes were 513, 550, and 489 nm after transport through porous media (Fig 14A, 14C and 14E).

The TEM image of ZnO NPs shows the uniform distribution of spherical-shaped NPs (Fig 14A–14F).

The increase in the size of TiO_2_ and ZnO NPs after transport was due to the agglomeration of nanoparticles. The differences in particle size of TiO_2_ and ZnO NPs for the tested pH 5, 7, and 9 were found to be insignificant before transport through porous media. The particle sizes of both NPs determined by TEM analysis were found to be in correlation with the DLS results. Even though the particles were found to be agglomerated after transport through the porous media, the morphology of NPs remained unchanged.

#### X-ray diffraction analysis

X-ray diffraction analysis was carried out to identify the crystal phases and to determine the crystalline size of material. The XRD pattern of TiO_2_ NPs (S1 Fig) shows the diffraction peaks at 25.2°, 37.81°, 47.9°, 54.0°, 54.9°, 62.6°, 68.8°, 70.3°, 75.2°, and 82.7°, which corresponds to (101), (004), (200), (105), (211), (204), (116), (220), (215), and (303) crystal planes. All diffraction peaks are well defined and perfectly matched with JCPDS card file no. 21–1272, and this confirms the presence of anatase as a predominant phase. Similar, X-ray diffraction were reported by Sadiq et al., [43] and Wei et al., [44]. The crystalline size of TiO_2_ NPs calculated from XRD patterns was 13.8 nm. Further, the X-ray diffraction analysis of ZnO NPs (S2 Fig) showed ten diffraction peaks at 31.7°, 34.3°, 36.2°, 47.5°, 56.5°, 62.8°, 66.3°, 67.9°, 69.0°, and 72.5°, assigned to (100), (002), (101), (102), (110), (103), (200), (112), (201), and (004) crystal planes. This result reveals the characteristic hexagonal wurtzite structure of ZnO NPs when compared with the card file no. 5–0664 of the JCPDS database [45]. The crystalline size of ZnO NPs calculated from XRD patterns was 53.09 nm.

Similarly, the X-ray diffraction analysis of TiO_2_ and ZnO NPs in presence of sand was also performed to observe the phase change of NPs. The XRD pattern of TiO2 and ZnO NPs in presence of sand shows the diffraction peak of TiO_2_ and ZnO NPs combined with the characteristic peaks of silica (at 20.9°), whereas, no overlapping of diffraction lines was observed between the two analytes. Thus, these results indicated that the crystalline shape of NPs was retained in the presence of sand (S3 and S4 Figs). The crystalline sizes of TiO2 NPs and ZnO NPs calculated from XRD patterns in presence of sand were 96.53 and 78.91 nm.

#### Fourier transform infrared spectroscopy

The FTIR analysis of TiO_2_ NPs were carried out and the data were compared to understand their behavior before and after transport through sand. The secondary derivative spectra of TiO_2_ NPs at pH 5 before transport (control) showed the presence of a sharp peak at 412 cm^-1^, corresponding to the stretching vibration of Ti–O–Ti [46]. The secondary derivative spectra of TiO_2_ NPs at pH 7 and 9 before transport also showed the presence of Ti–O–Ti stretching vibration peaks at 410 and 412 cm^-1^, as expected for TiO_2_ NPs. In contrast to the TiO_2_ NP spectrum before transport, the absence of Ti–O–Ti peak was observed in the spectra of TiO_2_ NPs after transport (at pH 5, 7, and 9), which indicates the absence/less release of TiO_2_ NPs as compared to the control (S5 Fig).

Similarly, the FTIR spectra was also recorded for ZnO NPs before and after transport at pH 5, 7, and 9. The secondary derivative spectra of ZnO NPs before transport (control) shows a significant peak around 417 cm^-1^ at pH 5 and 408 cm^-1^ at pH 7 and 9 that can be assigned to the vibration mode of Zn–O bonding [47, 48]. The presence of Zn-O bond at 417 cm^-1^ recorded for ZnO NPs after transport (at pH 5, 7, and 9) indicates the release of ZnO NPs (S6 Fig).

#### Conductivity Measurement

For both TiO_2_ NPs and ZnO NPs, the conductivity was found to increase with increase in ionic strength under all pH conditions (5, 7, and 9) (S8 and S9 Tables). However, in the case of ZnO NPs, the conductivity was more than TiO_2_ NPs. The conductivity was almost similar before and after TiO_2_ NP and ZnO NP transport in sand column.

## Conclusion

The current study suggests that the cotransport of TiO_2_ NPs and ZnO NPs in water-saturated porous media is much more complex than the transport of single nanoparticles. It also gives us a better understanding on the importance of cotransport of multicomponent nanoparticles as well as transport of individual nanoparticles in real aquatic environments, which are more complicated than those simulated in our study. Therefore, to better understand the cotransport behavior of TiO_2_ and ZnO NPs nanoparticles, further research is required to incorporate the effect of other factors (e.g., nanoparticle concentration and natural organic matter), which could influence the stability and interaction of nanoparticles.

## Supporting Information

S1 Fig XRD pattern of procured TiO_2_ nanoparticles. (TIF)

S2 Fig XRD pattern of procured ZnO nanoparticles. (TIF)

S3 Fig XRD pattern of procured TiO_2_ nanoparticles and sand. (TIF)

S4 Fig XRD pattern of procured ZnO nanoparticles and sand. (TIF)

S5 Fig FTIR spectra of TiO_2_ NPs before and after transport through sand at pH 5, 7.and 9. (JPG)

S6 Fig FTIR spectra of ZnO NPs before and after transport through sand at pH 5, 7.and 9. (TIF)

S1 Table Mean hydrodynamic size and zeta potential values of TiO_2_ NPs at different ionic strengths NaCl (0.1, 1, 10mM) and CaCl_2_ (0.1, 1, 10mM) and pH 5, 7 and 9. (DOCX)

S2 Table Mean hydrodynamic size and zeta potential values of ZnO NPs at different ionic strengths NaCl (0.1, 1, 10mM) and CaCl_2_ (0.1, 1, 10mM) and pH 5, 7 and 9. (DOCX)

S3 Table Zeta Potential values of fine sand in presence of NaCl (0.1, 1, 10mM) and CaCl_2_ (0.01, 0.05, 0.1mM) at pH 5, 7 and 9. (DOCX)

S4 Table Mass Balance of TiO_2_ NPs in different pH (5, 7 and 9mM) and ionic strengths (NaCl-0.1, 1, 10mM) conditions. (DOCX)

S5 Table Mass Balance of TiO_2_ NPs in different pH (5, 7 and 9) and ionic strengths CaCl_2_(0.01, 0.05, 0.1mM) conditions. (DOCX)

S6 Table Mass Balance of ZnO NPs in different pH (5, 7 and 9) and ionic strength (NaCl-0.1, 1, 10) conditions. (DOCX)

S7 Table Mass Balance of ZnO NPs in different pH (5, 7 and 9) and ionic strength CaCl_2_ (0.01, 0.05, 0.1) conditions. (DOCX)

S8 Table Conductivity of TiO_2_ NPs solution at different pH (5, 7 and 9) and ionic strengths (NaCl-0.1, 1, 10; CaCl_2_-0.01, 0.05, 0.1). (DOCX)

S9 Table Conductivity of ZnO NPs solution at different pH (5, 7 and 9) and ionic strength (NaCl-0.1, 1, 10; CaCl_2_-0.01, 0.05, 0.1). (DOCX)
