# Supplementary figures and images for: Individual and Co Transport Study of Titanium Dioxide NPs and Zinc Oxide NPs in Porous Media
Source: PLoS One. 2015 Aug 7;10(8):e0134796. doi: 10.1371/journal.pone.0134796 (PMC4529095; doi:10.1371/journal.pone.0134796)

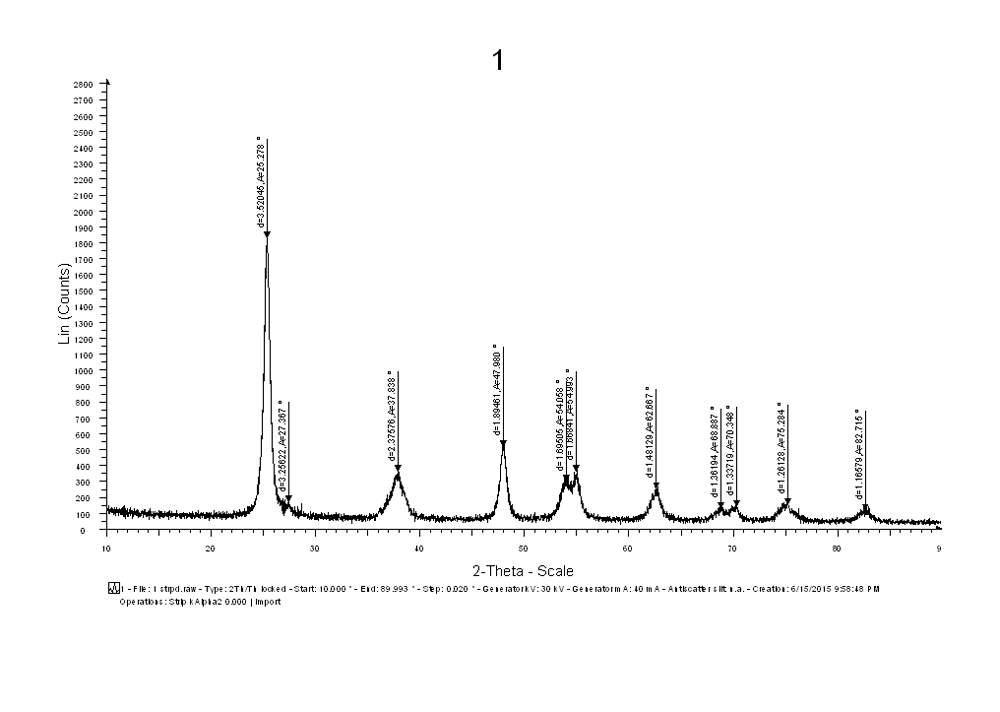

Supplement: S1 Fig — (TIF) [file pone.0134796.s001.tif]

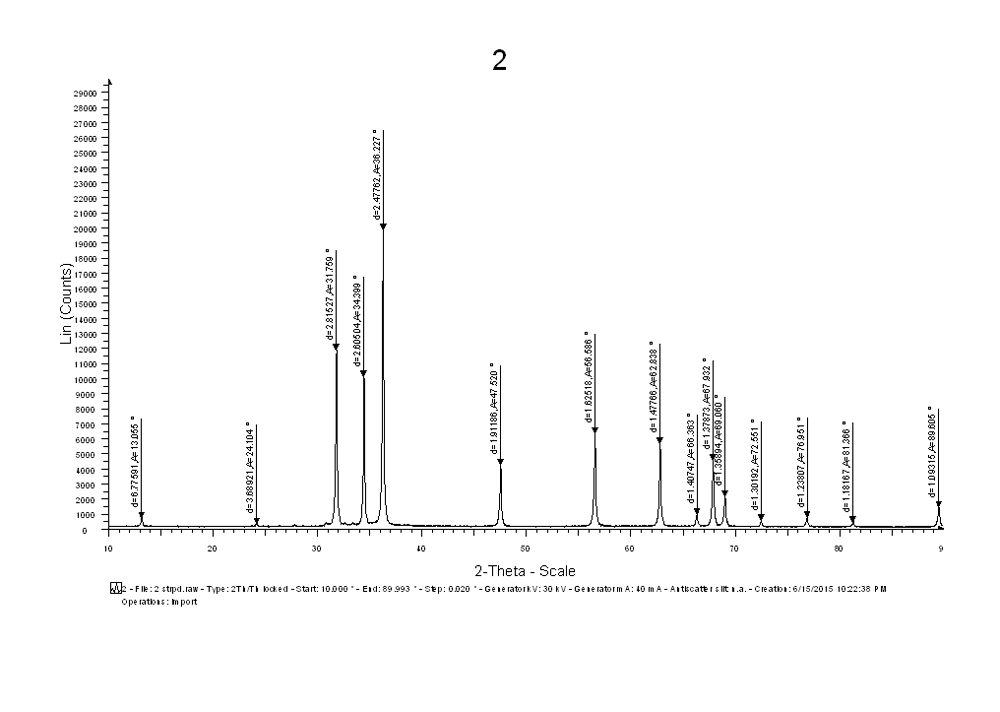

Supplement: S2 Fig — (TIF) [file pone.0134796.s002.tif]

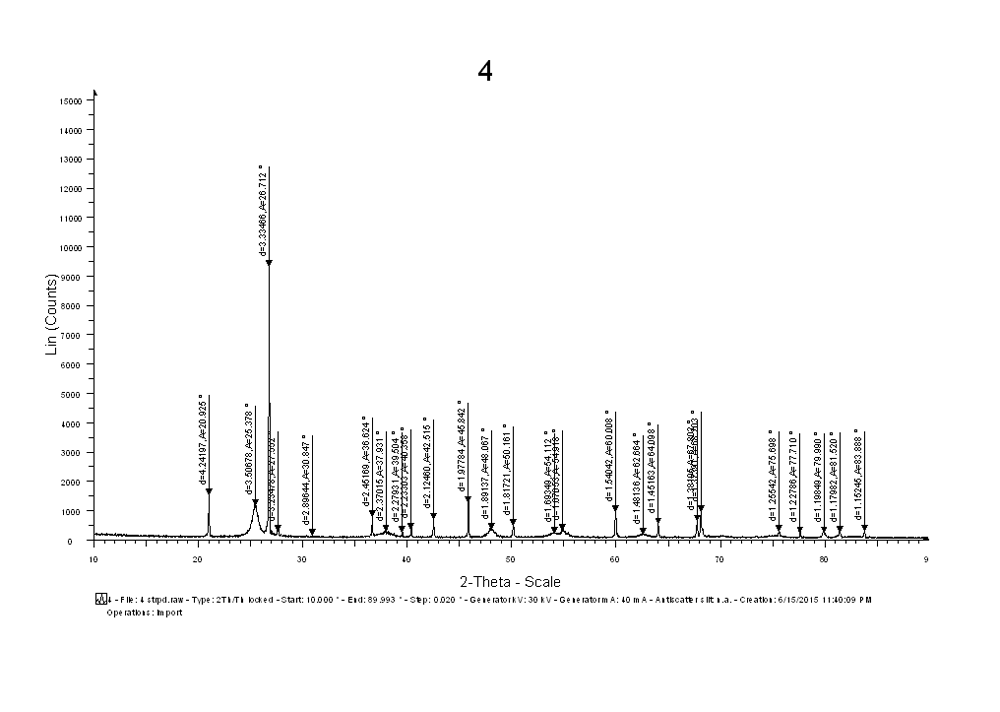

Supplement: S3 Fig — (TIF) [file pone.0134796.s003.tif]

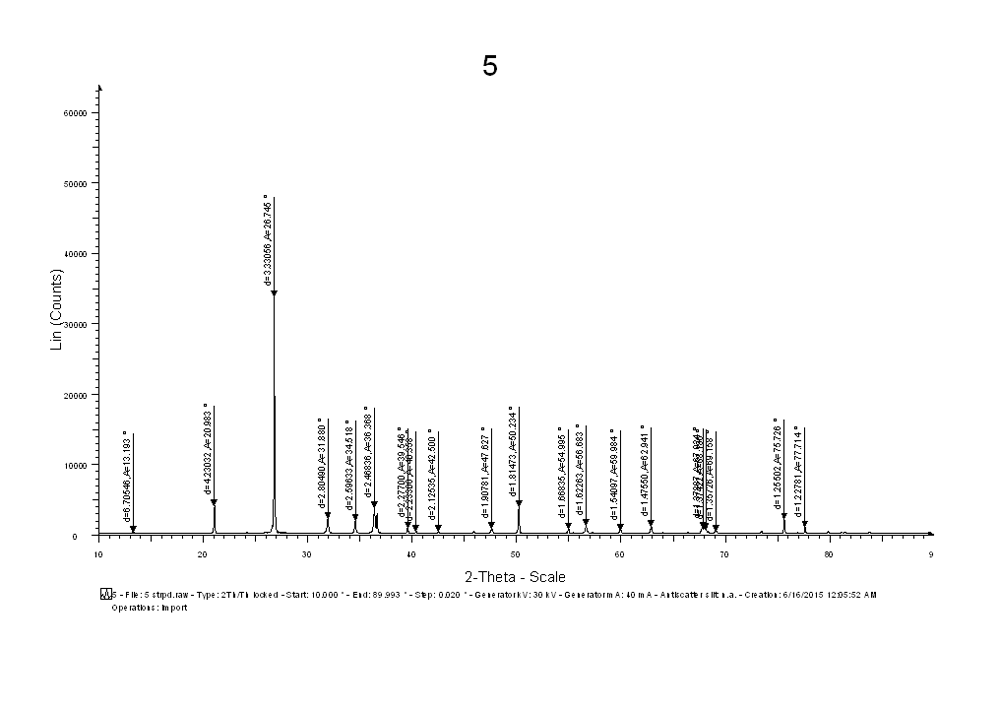

Supplement: S4 Fig — (TIF) [file pone.0134796.s004.tif]

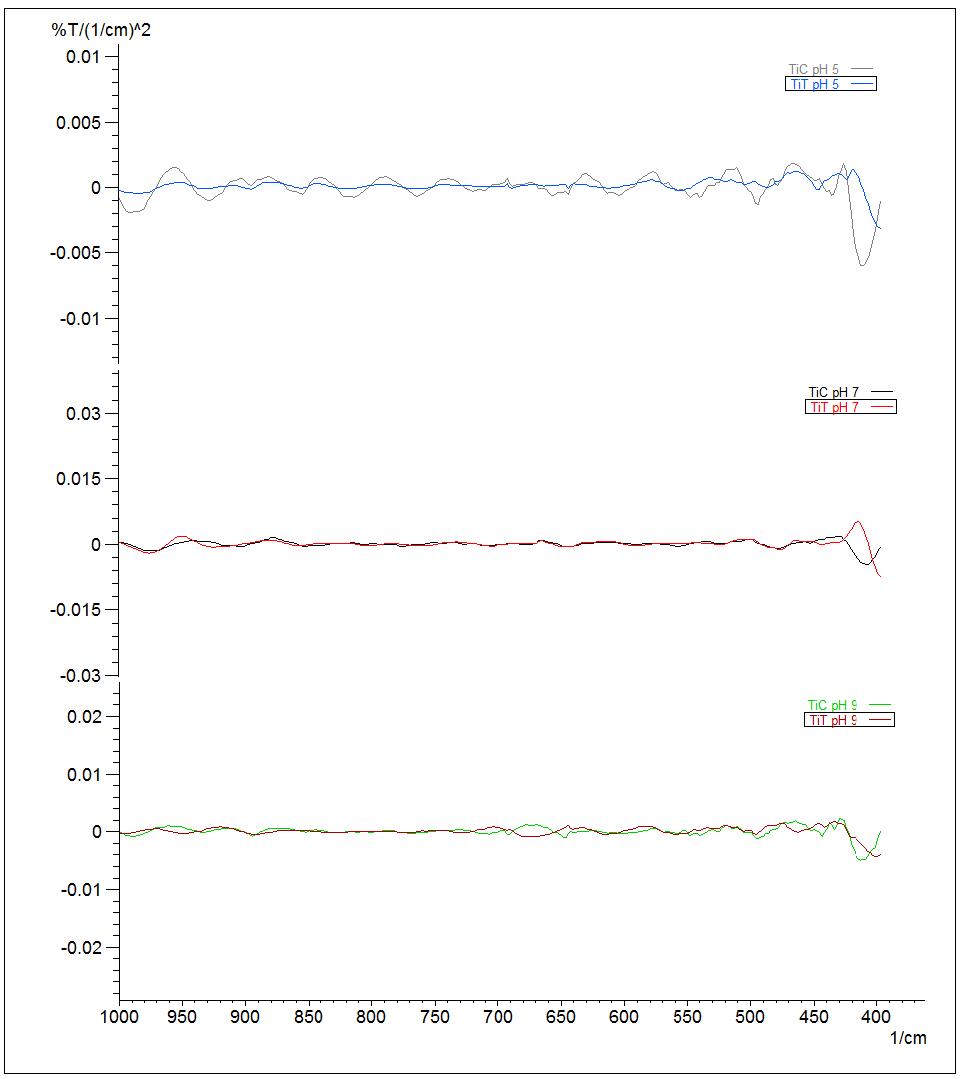

Supplement: S5 Fig — (JPG) [file pone.0134796.s005.jpg]

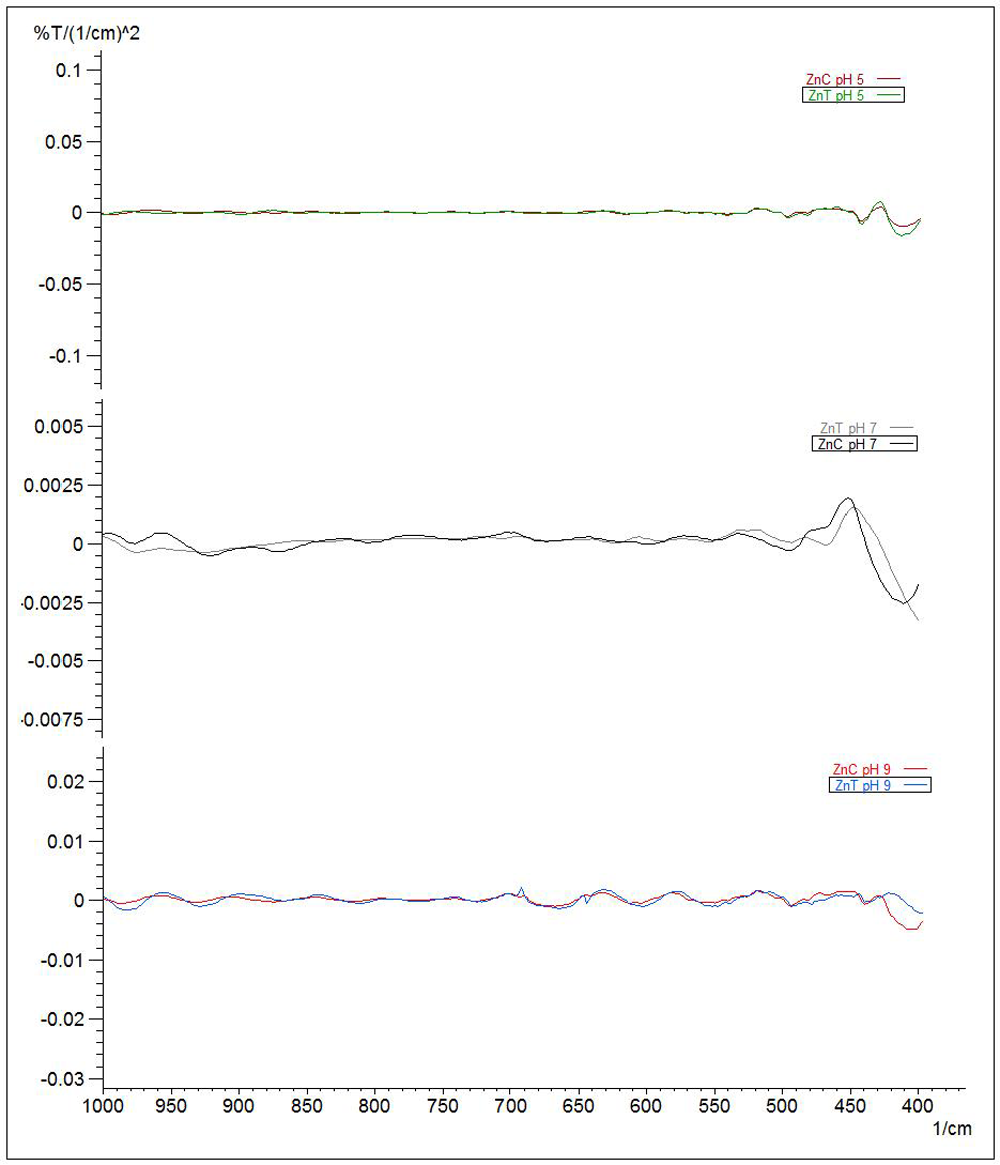

Supplement: S6 Fig — (TIF) [file pone.0134796.s006.tif]
