# Supplementary material for: Individual and Co Transport Study of Titanium Dioxide NPs and Zinc Oxide NPs in Porous Media
Source: PLoS One. 2015 Aug 7;10(8):e0134796. doi: 10.1371/journal.pone.0134796 (PMC4529095; doi:10.1371/journal.pone.0134796)
Supplement: S1 Table — (DOCX) [file pone.0134796.s007.docx]

| **pH** | **Salt Type** | **Ionic Strength (mM)** | **Zeta Potential**  **(mV)** | **Mean Hydrodynamic Diameter**  **(nm)** |
| --- | --- | --- | --- | --- |
| **5** | **NaCl** | 0.1 | 23±1.41 | 483.8±4.1 |
|  |  | 1 | 23.5±0.70 | 647.3±9.0 |
|  |  | 10 | 21±1.41 | 947.2±3.8 |
|  | **CaCl_2_** | 0.01 | 26± 1.41 | 421.5±2.5 |
|  |  | 0.05 | 24±1.41 | 441.6±7.2 |
|  |  | 0.1 | 21.5±0.70 | 527.8±7.9 |
| **7** | **NaCl** | 0.1 | -22.5±0.70 | 431.6±3.4 |
|  |  | 1 | -19±1.41 | 524.9±3.3 |
|  |  | 10 | -14±2.82 | 774.3±5.7 |
|  | **CaCl_2_** | 0.01 | -22±2.82 | 446.8±5.1 |
|  |  | 0.05 | -19±1.41 | 491.5±4.0 |
|  |  | 0.1 | -17±2.82 | 537.2±5.6 |
| 9 | **NaCl** | 0.1 | -31±1.41 | 468.0±9.4 |
|  |  | 1 | -28.5±2.12 | 535.7±1.7 |
|  |  | 10 | -23.5±2.12 | 831.4±6.1 |
|  | **CaCl_2_** | 0.01 | -31±5.65 | 493.6±4.25 |
|  |  | 0.05 | -29±2.82 | 564.4±2.19 |
|  |  | 0.1 | -27±7.07 | 772.9±8.2 |

**S1 Table. Mean hydrodynamic size and zeta potential values of TiO_2_ NPs at different ionic strengths NaCl (0.1, 1, 10mM) and CaCl_2_ (0.1, 1, 10mM) and pH 5, 7 and 9.**
