# Supplementary material for: Individual and Co Transport Study of Titanium Dioxide NPs and Zinc Oxide NPs in Porous Media
Source: PLoS One. 2015 Aug 7;10(8):e0134796. doi: 10.1371/journal.pone.0134796 (PMC4529095; doi:10.1371/journal.pone.0134796)
Supplement: S2 Table — (DOCX) [file pone.0134796.s008.docx]

| **pH** | **Salt Type** | **Ionic Strength (mM)** | **Zeta Potential**  **(mV)** | **Mean Hydrodynamic Diameter**  **(nm)** |
| --- | --- | --- | --- | --- |
| **5** | **NaCl** | 0.1 | 24±5.65 | 330.6±3.2 |
|  |  | 1 | 23.5±4.94 | 449.8±7.5 |
|  |  | 10 | 22.5±3.53 | 613.4±2.0 |
|  | **CaCl_2_** | 0.01 | 26.5±2.12 | 306.1±2.1 |
|  |  | 0.05 | 24±5.65 | 391.7±1.0 |
|  |  | 0.1 | 20.5±0.70 | 491.0±2.1 |
| **7** | **NaCl** | 0.1 | 17±2.82 | 306.9±1.5 |
|  |  | 1 | 14±5.65 | 420.1±5.2 |
|  |  | 10 | 12±2.82 | 623.5±1.9 |
|  | **CaCl_2_** | 0.01 | 16.5±7.94 | 321.7±4.9 |
|  |  | 0.05 | 15.5±3.53 | 356.9±2.82 |
|  |  | 0.1 | 11.5±3.53 | 492.5±3.4 |
| 9 | **NaCl** | 0.1 | -22±7.07 | 373.4±3.3 |
|  |  | 1 | -18±2.82 | 471.7±5.01 |
|  |  | 10 | -13±4.24 | 572.0±1.1 |
|  | **CaCl_2_** | 0.01 | -21.5±3.53 | 370.0±4.7 |
|  |  | 0.05 | -19±1.41 | 490.4±3.5 |
|  |  | 0.1 | -17±2.82 | 604.0±4.1 |

**S2 Table. Mean hydrodynamic size and zeta potential values of ZnO NPs at different ionic strengths NaCl (0.1, 1, 10mM) and CaCl_2_ (0.1, 1, 10mM) and pH 5, 7 and 9.**
