# Supplementary material for: Individual and Co Transport Study of Titanium Dioxide NPs and Zinc Oxide NPs in Porous Media
Source: PLoS One. 2015 Aug 7;10(8):e0134796. doi: 10.1371/journal.pone.0134796 (PMC4529095; doi:10.1371/journal.pone.0134796)
Supplement: S3 Table — (DOCX) [file pone.0134796.s009.docx]

**S3 Table. Zeta Potential values of fine sand in presence of NaCl (0.1, 1, 10mM) and CaCl_2_ (0.01, 0.05, 0.1mM) at pH 5, 7 and 9.**

| **pH** | **Salt Type** | **Ionic Strength (mM)** | **Zeta Potential of sand**  **(mV)** |
| --- | --- | --- | --- |
| **5** | **NaCl** | 0.1 | -53.9± 1.18 |
|  |  | 1 | -45.3±1.95 |
|  |  | 10 | -42.7±1.12 |
|  | **CaCl_2_** | 0.01 | -33.3±1.73 |
|  |  | 0.05 | -31.9±1.46 |
|  |  | 0.1 | -30.6±2.02 |
| **7** | **NaCl** | 0.1 | -67.1±2.12 |
|  |  | 1 | -63.7±3.51 |
|  |  | 10 | -56.3±1.76 |
|  | **CaCl_2_** | 0.01 | -53.3±3.6 |
|  |  | 0.05 | -44.3±0.95 |
|  |  | 0.1 | -42.6±1.52 |
| 9 | **NaCl** | 0.1 | -72.7±2.17 |
|  |  | 1 | -69.1±2.16 |
|  |  | 10 | -68±2.52 |
|  | **CaCl_2_** | 0.01 | -57.6±0.54 |
|  |  | 0.05 | -54.3±3.60 |
|  |  | 0.1 | -50.7±1.50 |
