# Supplementary material for: Individual and Co Transport Study of Titanium Dioxide NPs and Zinc Oxide NPs in Porous Media
Source: PLoS One. 2015 Aug 7;10(8):e0134796. doi: 10.1371/journal.pone.0134796 (PMC4529095; doi:10.1371/journal.pone.0134796)
Supplement: S4 Table — (DOCX) [file pone.0134796.s010.docx]

| **Sample** | **pH** | **Ionic strength (mM)** | **Solution Component** | **Mass Balance** | |
| --- | --- | --- | --- | --- | --- |
|  |  |  |  | **% eff** | **% rec** |
| TiO_2_ | 5 | 0.1 | NaCl, w/o ZnO | 18.4 | 81.6 |
|  |  |  | NaCl, w/ 5 mg L^-1^ ZnO | 19.1 | 79.9 |
|  |  | 1 | NaCl, w/o ZnO | 14.2 | 86.6 |
|  |  |  | NaCl, w/ 5 mg L^-1^ ZnO | 15.8 | 83.1 |
|  |  | 10 | NaCl, w/o ZnO | 9.4 | 91.7 |
|  |  |  | NaCl, w/ 5 mg L^-1^ ZnO | 10.8 | 85 |
|  | 7 | 0.1 | NaCl, w/o ZnO | 80.4 | 89.3 |
|  |  |  | NaCl, w/ 5 mg L^-1^ ZnO | 53.7 | 81.6 |
|  |  | 1 | NaCl, w/o ZnO | 33.2 | 95.2 |
|  |  |  | NaCl, w/ 5 mg L^-1^ ZnO | 28.4 | 24.8 |
|  |  | 10 | NaCl, w/o ZnO | 11.3 | 87.3 |
|  |  |  | NaCl, w/ 5 mg L^-1^ ZnO | 11 | 97.5 |
|  | 9 | 0.1 | NaCl, w/o ZnO | 101.4 | 105.1 |
|  |  |  | NaCl, w/ 5 mg L^-1^ ZnO | 100 | 108.1 |
|  |  | 1 | NaCl, w/o ZnO | 50 | 112.1 |
|  |  |  | NaCl, w/ 5 mg L^-1^ ZnO | 71.9 | 114.1 |
|  |  | 10 | NaCl, w/o ZnO | 20 | 102.8 |
|  |  |  | NaCl, w/ 5 mg L^-1^ ZnO | 23.1 | 106 |

**S4 Table. Mass Balance of TiO_2_ NPs in different pH (5, 7 and 9mM) and ionic strengths (NaCl-0.1, 1, 10mM) conditions.**

**^% eff^ : Percentage of nanoparticle eluted out from column**

**^% rec :^ Percentage of nanoparticle recovered inside the column**
