# Supplementary material for: Individual and Co Transport Study of Titanium Dioxide NPs and Zinc Oxide NPs in Porous Media
Source: PLoS One. 2015 Aug 7;10(8):e0134796. doi: 10.1371/journal.pone.0134796 (PMC4529095; doi:10.1371/journal.pone.0134796)
Supplement: S5 Table — (DOCX) [file pone.0134796.s011.docx]

| **Sample** | **pH** | **Ionic strength (mM)** | **Solution Component** | **Mass Balance** | |
| --- | --- | --- | --- | --- | --- |
|  |  |  |  | **% eff** | **%rec** |
| TiO_2_ | 5 | 0.1 | CaCl_2_, w/o ZnO | 17.04 | 81.6 |
|  |  |  | CaCl_2_, w/ 5 mg L^-1^ ZnO | 17.7 | 79.9 |
|  |  | 1 | CaCl_2_, w/o ZnO | 10.1 | 86.6 |
|  |  |  | CaCl_2_, w/ 5 mg L^-1^ ZnO | 11.8 | 83.1 |
|  |  | 10 | CaCl_2_, w/o ZnO | 7.3 | 91.7 |
|  |  |  | CaCl_2_, w/ 5 mg L^-1^ ZnO | 7.8 | 85 |
|  | 7 | 0.1 | CaCl_2_, w/o ZnO | 78.7 | 86.8 |
|  |  |  | CaCl_2_, w/ 5 mg L^-1^ ZnO | 66.2 | 76 |
|  |  | 1 | CaCl_2_, w/o ZnO | 30.4 | 100.4 |
|  |  |  | CaCl_2_, w/ 5 mg L^-1^ ZnO | 28.1 | 102.6 |
|  |  | 10 | CaCl_2_, w/o ZnO | 12 | 108.2 |
|  |  |  | CaCl_2_, w/ 5 mg L^-1^ ZnO | 9.2 | 107.8 |
|  | 9 | 0.1 | CaCl_2_, w/o ZnO | 95.7 | 105.4 |
|  |  |  | CaCl_2_, w/ 5 mg L^-1^ ZnO | 109.4 | 108.1 |
|  |  | 1 | CaCl_2_, w/o ZnO | 37.1 | 106.6 |
|  |  |  | CaCl_2_, w/ 5 mg L^-1^ ZnO | 44 | 109.1 |
|  |  | 10 | CaCl_2_, w/o ZnO | 19 | 107.1 |
|  |  |  | CaCl_2_, w/ 5 mg L^-1^ ZnO | 22.6 | 110.8 |

**S5 Table. Mass Balance of TiO_2_ NPs in different pH (5, 7 and 9) and ionic strengths CaCl_2_(0.01, 0.05, 0.1mM) conditions.**

**^% eff^ : Percentage of nanoparticle eluted out from column**

**^% rec :^ Percentage of nanoparticle recovered inside the column**
