# Supplementary material for: Individual and Co Transport Study of Titanium Dioxide NPs and Zinc Oxide NPs in Porous Media
Source: PLoS One. 2015 Aug 7;10(8):e0134796. doi: 10.1371/journal.pone.0134796 (PMC4529095; doi:10.1371/journal.pone.0134796)
Supplement: S6 Table — (DOCX) [file pone.0134796.s012.docx]

| **Sample** | **pH** | **Ionic strength (mM)** | **Solution Component** | **Mass Balance** | |
| --- | --- | --- | --- | --- | --- |
|  |  |  |  | **% eff** | **%rec** |
| ZnO | 5 | 0.1 | NaCl, w/o TiO_2_ | 19 | 81 |
|  |  |  | NaCl, w/ 10 mg L^-1^ TiO_2_ | 15.8 | 85.8 |
|  |  | 1 | NaCl, w/o TiO_2_ | 11 | 89.5 |
|  |  |  | NaCl, w/ 10 mg L^-1^ TiO_2_ | 9.6 | 91.2 |
|  |  | 10 | NaCl, w/o TiO_2_ | 6 | 93.4 |
|  |  |  | NaCl, w/ 10 mg L^-1^ TiO_2_ | 6.4 | 94.7 |
|  | 7 | 0.1 | NaCl, w/o TiO_2_ | 13.1 | 87.6 |
|  |  |  | NaCl, w/ 10 mg L^-1^ TiO_2_ | 19 | 81 |
|  |  | 1 | NaCl, w/o TiO_2_ | 10.2 | 91 |
|  |  |  | NaCl, w/ 10 mg L^-1^ TiO_2_ | 13.9 | 87.1 |
|  |  | 10 | NaCl, w/o TiO_2_ | 6.7 | 93.4 |
|  |  |  | NaCl, w/ 10 mg L^-1^ TiO_2_ | 9.6 | 91.4 |
|  | 9 | 0.1 | NaCl, w/o TiO_2_ | 86.5 | 90.0 |
|  |  |  | NaCl, w/ 10 mg L^-1^ TiO_2_ | 93 | 95.6 |
|  |  | 1 | NaCl, w/o TiO_2_ | 77.6 | 84.4 |
|  |  |  | NaCl, w/ 10 mg L^-1^ TiO_2_ | 86.8 | 91.2 |
|  |  | 10 | NaCl, w/o TiO_2_ | 68 | 79.9 |
|  |  |  | NaCl, w/ 10 mg L^-1^ TiO_2_ | 67.4 | 73.8 |

**S6 Table. Mass Balance of ZnO NPs in different pH (5, 7 and 9) and ionic strength (NaCl-0.1, 1, 10) conditions.**

**^% eff^ : Percentage of nanoparticle eluted out from column**

**^% rec :^ Percentage of nanoparticle recovered inside the column**
