# Supplementary material for: Individual and Co Transport Study of Titanium Dioxide NPs and Zinc Oxide NPs in Porous Media
Source: PLoS One. 2015 Aug 7;10(8):e0134796. doi: 10.1371/journal.pone.0134796 (PMC4529095; doi:10.1371/journal.pone.0134796)
Supplement: S7 Table — (DOCX) [file pone.0134796.s013.docx]

| **Sample** | **pH** | **Ionic strength (mM)** | **Solution Component** | **Mass Balance** | |
| --- | --- | --- | --- | --- | --- |
|  |  |  |  | **% eff** | **%rec** |
| ZnO | 5 | 0.1 | CaCl_2_, w/o TiO_2_ | 20.4 | 83.6 |
|  |  |  | CaCl_2_, w/ 10 mg L^-1^ TiO_2_ | 17.9 | 86.1 |
|  |  | 1 | CaCl_2_, w/o TiO_2_ | 12.3 | 88.2 |
|  |  |  | CaCl_2_, w/ 10 mg L^-1^ TiO_2_ | 10.1 | 90.5 |
|  |  | 10 | CaCl_2_, w/o TiO_2_ | 5.6 | 85.2 |
|  |  |  | CaCl_2_, w/ 10 mg L^-1^ TiO_2_ | 4.7 | 102.4 |
|  | 7 | 0.1 | CaCl_2_, w/o TiO_2_ | 12.5 | 88.6 |
|  |  |  | CaCl_2_, w/ 10 mg L^-1^ TiO_2_ | 14.8 | 85.9 |
|  |  | 1 | CaCl_2_, w/o TiO_2_ | 9.8 | 92.2 |
|  |  |  | CaCl_2_, w/ 10 mg L^-1^ TiO_2_ | 10.2 | 89.4 |
|  |  | 10 | CaCl_2_, w/o TiO_2_ | 6.7 | 93 |
|  |  |  | CaCl_2_, w/ 10 mg L^-1^ TiO_2_ | 8 | 91 |
|  | 9 | 0.1 | CaCl_2_, w/o TiO_2_ | 82.2 | 91.3 |
|  |  |  | CaCl_2_, w/ 10 mg L^-1^ TiO_2_ | 90.3 | 95.7 |
|  |  | 1 | CaCl_2_, w/o TiO_2_ | 71.7 | 82.5 |
|  |  |  | CaCl_2_, w/ 10 mg L^-1^ TiO_2_ | 78.2 | 85.7 |
|  |  | 10 | CaCl_2_, w/o TiO_2_ | 56.7 | 68.9 |
|  |  |  | CaCl_2_, w/ 10 mg L^-1^ TiO_2_ | 67.4 | 63.7 |

**S7 Table. Mass Balance of ZnO NPs in different pH (5, 7 and 9) and ionic strength CaCl2 (0.01, 0.05, 0.1) conditions.**

**^% eff^ : Percentage of nanoparticle eluted out from column**

**^% rec :^ Percentage of nanoparticle recovered inside the column**
