# Supplementary material for: Individual and Co Transport Study of Titanium Dioxide NPs and Zinc Oxide NPs in Porous Media
Source: PLoS One. 2015 Aug 7;10(8):e0134796. doi: 10.1371/journal.pone.0134796 (PMC4529095; doi:10.1371/journal.pone.0134796)
Supplement: S8 Table — (DOCX) [file pone.0134796.s014.docx]

**S8 Table. Conductivity of TiO_2_ NPs solution at different pH (5, 7 and 9) and ionic strengths (NaCl-0.1, 1, 10; CaCl_2_-0.01, 0.05, 0.1).**

| **pH** | **Salt Type** | **Ionic Strength (mM)** | **Conductivity**  **(micromho/cm)** |
| --- | --- | --- | --- |
| **5** | **NaCl** | 0.1 | 2.1±0.33 |
|  |  | 1 | 2.3±0.65 |
|  |  | 10 | 2.6±0.45 |
|  | **CaCl_2_** | 0.01 | 2.2±0.45 |
|  |  | 0.05 | 2.4±0.42 |
|  |  | 0.1 | 2.6±.031 |
| **7** | **NaCl** | 0.1 | 2.2±0.27 |
|  |  | 1 | 2.4±0.35 |
|  |  | 10 | 2.6±0.56 |
|  | **CaCl_2_** | 0.01 | 2.4±0.69 |
|  |  | 0.05 | 2.6±.033 |
|  |  | 0.1 | 2.7±.023 |
| 9 | **NaCl** | 0.1 | 2.3±0.36 |
|  |  | 1 | 2.4±0.49 |
|  |  | 10 | 2.8±0.37 |
|  | **CaCl_2_** | 0.01 | 2.5±0.42 |
|  |  | 0.05 | 2.6±0.34 |
|  |  | 0.1 | 2.8±0.83 |
