# Supplementary material for: Individual and Co Transport Study of Titanium Dioxide NPs and Zinc Oxide NPs in Porous Media
Source: PLoS One. 2015 Aug 7;10(8):e0134796. doi: 10.1371/journal.pone.0134796 (PMC4529095; doi:10.1371/journal.pone.0134796)
Supplement: S9 Table — (DOCX) [file pone.0134796.s015.docx]

**S9 Table. Conductivity of ZnO NPs solution at different pH (5, 7 and 9) and ionic strength (NaCl-0.1, 1, 10; CaCl_2_-0.01, 0.05, 0.1).**

| **pH** | **Salt Type** | **Ionic Strength (mM)** | **Conductivity**  **(micromho/cm)** |
| --- | --- | --- | --- |
| **5** | **NaCl** | 0.1 | 3.3±0.84 |
|  |  | 1 | 3.5±0.38 |
|  |  | 10 | 3.8±0.83 |
|  | **CaCl_2_** | 0.01 | 3.5±0.95 |
|  |  | 0.05 | 3.6±0.52 |
|  |  | 0.1 | 3.9±0.53 |
| **7** | **NaCl** | 0.1 | 3.4±0.68 |
|  |  | 1 | 3.6±0.42 |
|  |  | 10 | 3.9±0.46 |
|  | **CaCl_2_** | 0.01 | 3.7±0.36 |
|  |  | 0.05 | 3.8±0.75 |
|  |  | 0.1 | 3.9±0.37 |
| 9 | **NaCl** | 0.1 | 3.5±0.28 |
|  |  | 1 | 3.7±0.47 |
|  |  | 10 | 4.0±0.28 |
|  | **CaCl_2_** | 0.01 | 3.9±0.49 |
|  |  | 0.05 | 4.0±0.39 |
|  |  | 0.1 | 4.1±0.26 |
